# Development of an In Vitro Test Method to Replace an Animal-Based Potency Test for Pertactin Antigen in Multivalent Vaccines

**DOI:** 10.3390/vaccines11020275

**Published:** 2023-01-27

**Authors:** Jason Szeto, Aruun Beharry, Tricia Chen, Eric Zholumbetov, Emilie Daigneault, Marin Ming, Iain Lounsbury, Nelson Eng, Nemika Thangavadivel, Robbie Jin, Aurélie Denis-Jacquot, Bahram Behnam Azad, Meili Li, Diana Keizner, Marcus Liu, Sophia S. F. Lee, Kai He, Beata Gajewska

**Affiliations:** 1 Analytical Sciences Immunology, Sanofi, 1755 Steeles Avenue West, Toronto, ON M2R 3T4, Canada; 2 Medical Science Program, Medical Sciences Building Room M138, Western University, London, ON N6A 5C1, Canada; 3 Quality Control Immunochemistry, Sanofi, 1755 Steeles Avenue West, Toronto, ON M2R 3T4, Canada; 4 Quality Control Analytical Excellence, Sanofi, 1755 Steeles Avenue West, Toronto, ON M2R 3T4, Canada; 5 Department of Biology, University of Toronto Scarborough, 1265 Military Trail, Toronto, ON M1C 1A4, Canada; 6 Nonclinical Biostatistics, Sanofi, 1755 Steeles Avenue West, Toronto, ON M2R 3T4, Canada; 7 Quality Control Analytical Excellence Biostatistics, Sanofi, 1755 Steeles Avenue West, Toronto, ON M2R 3T4, Canada; 8 Analytical Sciences North America, Sanofi, 1755 Steeles Avenue West, Toronto, ON M2R 3T4, Canada

**Keywords:** antigenicity, pertactin, ELISA, replacing animal testing, in vitro testing, 3Rs, vaccines

## Abstract

There is increasing interest to replace animal-based potency assays used routinely to test vaccines, since they are highly variable, are costly, and present ethical concerns. The development of relevant in vitro assays is part of the solution. Using pertactin (PRN) antigen as an example in DTaP-IPV (diphtheria, tetanus, acellular pertussis, and inactivated poliovirus) vaccines, a PRN antigenicity ELISA was developed using two monoclonal antibodies with a high affinity to unique PRN epitopes, relevance to human immune responses, and evidence of functionality. The ELISA measured consistent PRN antigenicity between the vaccine lots and was validated to demonstrate its accuracy, precision, linearity, and specificity. Notably, the PRN antigenicity ELISA was more sensitive than the mouse-based potency test and could more effectively differentiate between degraded and intact vaccine lots compared to the in vivo test. From these studies, the PRN antigenicity ELISA is proposed as an in vitro replacement for the in vivo potency test for PRN in DTaP-IPV-based formulations. Important considerations in this study included comprehensive antibody characterization, testing of multiple vaccine lots, method validation, and comparison to animal-based potency. Together, these factors form part of an overall strategy that ensures reliable and relevant in vitro assays are developed to replace animal tests.

## 1. Introduction

As part of routine potency testing for vaccine product release, manufacturers continue to rely on animal-based test methods, particularly for well-established vaccines that have been on the market for decades. Such resource-intensive methods have proven to be challenging and present issues, including high variability, high cost, and ethical concerns over animal usage [1]. Due to the variability of animal-based tests, there is a risk of delaying the release of, or rejecting, a vaccine batch that is fully potent, thus impacting batch release and vaccine supply to the market.

The ‘3Rs’ principle encourages the Replacement, Reduction, and Refinement of animal testing [2]. Unlike many legacy vaccines, modern vaccines increasingly rely upon the use of in vitro analytical test methods to release products. There remains much opportunity to implement in vitro tests to replace animal-based potency tests that are currently used for established legacy vaccines, including multivalent diphtheria, tetanus, and acellular pertussis (DTaP) vaccines that have been a mainstay of recommended immunization schedules in many countries. 

An example is DTaP-IPV, a complex vaccine containing diphtheria toxoid, tetanus toxoid, three inactivated poliovirus types (IPV), and acellular pertussis (aP) antigens consisting of pertactin, pertussis toxoid, filamentous hemagglutinin, and fimbriae types 2 and 3, in the presence of aluminum phosphate (AlPO_4_) adjuvant. When this vaccine is used to resuspend a lyophilized Act-Hib component (purified polyribosyl-ribitol phosphate of *Haemophilus influenzae b* covalently bound to a tetanus toxoid conjugate), the resulting product is referred to as reconstituted DTaP-IPV/Hib product.

An animal-based potency test applied to DTaP-IPV and DTaP-IPV/Hib is the in vivo acellular pertussis mouse immunogenicity assay. In general, this test involves immunizing groups of mice via the intraperitoneal route with dilutions of the test vaccine and a control vaccine. The mice are bled after four weeks, and the serum antibodies against each pertussis antigen are measured by using an ELISA test. The criteria of acceptance are based on both the quantity of antibody response against each pertussis antigen and the number of responding mice. This test utilizes a significant number of animals each year; however, like many animal-based tests, it is inherently variable and subjected to repeat testing, which can lead to increased delays in releasing vaccines to the market and possibly false rejection of batches unrelated to product quality, which can contribute to vaccine shortages.

A key antigen in the acellular pertussis vaccines described above is pertactin (PRN). This protein is expressed on the surface of *Bordetella pertussis*, the causative agent of whooping cough. PRN is a 60 kDa type I autotransporter, which is processed from a 93 kDa large precursor to a smaller 60.4 kDa portion [3]. The crystal structure of the first 539 amino acids of PRN has been determined, showing that the protein forms a 16-stranded parallel β-helix with a V-shaped cross section [4]. Several loops protrude from the helix which contains a) the RGD-domain motif (Arg-Gly-Asp) [4] characterized as a cell attachment site in various mammalian adhesion molecules, such as fibronectin, vitronectin, fibrinogen, and van Willebrand factor [5], and b) two proline-rich regions (PRR) [4], which occur widely in proteins with binding activity. Several studies support a role of PRN in the adhesion and invasion of bacteria through its RGD motif [6,7,8,9]. PRN appears to be the only antigen in acellular pertussis vaccines that can generate a bactericidal antibody response, and this may, in part, be responsible for the rise in circulating PRN-deficient *B. pertussis* strains due to the selective pressure from the acellular vaccination [10].

Here, we describe the development of a sandwich antigenicity ELISA as a candidate in vitro assay to replace the mouse-based assay that is currently used to determine the potency of PRN antigen in DTaP-IPV and DTaP-IPV/Hib. Antigenicity is a measurement of epitope presentation and intactness, assessed through the binding of specific antibodies to an antigen. The identification of functional, immunologically relevant antibodies for use in antigenicity testing is a key step in developing an in vitro replacement for an in vivo potency test method. Our group has previously characterized a panel of murine anti-PRN monoclonal antibodies (mAbs) to identify suitable candidates for developing in vitro assays [11,12]. Among these, two mAb clones (designated mAbs 3–4 and 3–5) were shown to bind to the surface of a relatively high proportion of *B. pertussis* bacteria, compared to the other anti-PRN mAbs studied, using a flow cytometry assay [11]. These anti-PRN mAbs were able to significantly reduce the binding of PRN-coated latex beads to THP-1 monocytic cells, supporting a role of PRN in host cell adhesion [11]. Furthermore, the PRN epitopes for mAbs 3–4 and 3–5 were identified using a hydrogen/deuterium exchange mass spectrometry (HDX-MS) and were shown to be located in different regions of the antigen [12]. mAb 3–4 recognizes a conformational epitope covering amino acids (aa) 420–436, 455–464, 477–492, and 505–517 of PRN, while mAb 3–5 recognizes an epitope spanning aa 234–244. The epitope for mAb 3–5 is considered to be conformational since the binding of this antibody was lost upon heat denaturation of PRN, and this mAb did not bind to purified aa 234–244 peptide fragment alone [12].

The data from the above studies [11,12] and additional antibody characterization data described in this study were used to select anti-PRN mAbs 3–4 and 3–5 for the development of the PRN antigenicity ELISA. The ELISA was validated to demonstrate its accuracy, precision, linearity, and specificity for PRN in the vaccine formulation. Importantly, the ELISA was more sensitive than the animal-based potency test for PRN and was able to more effectively differentiate between intact and variously degraded vaccine lots compared to the in vivo test. Based on these studies, the PRN antigenicity ELISA is proposed as a viable in vitro replacement of the in vivo potency test for PRN in DTaP-IPV and DTaP-IPV/Hib vaccine formulations.

## 2. Materials and Methods

### 2.1. Anti-Pertactin (PRN) Monoclonal Antibodies (mAbs) 

The generation of the murine anti-PRN mAb clones 3–1, 3–3, 3–4, 3–5, 3–16, and 3–21 has been described previously [11].

### 2.2. Biolayer Interferometry

The affinities of the anti-PRN mAbs for the purified PRN antigen were assessed using a biolayer interferometry (BLI) (Octet 384 system) (Sartorius, Goettingen, Germany). Anti-mouse IgG Fc capture biosensors (Sartorius, Goettingen, Germany) were used to capture individual anti-PRN mAbs at 20 μg/mL. The purified PRN antigen was diluted to 20 μg/mL in PBS, and a series of 3-fold dilutions from this starting concentration were added to the assay plate. The interaction of the sensor-captured mAbs with the diluted PRN antigen series was monitored by the BLI using an association time of 300 s, followed by a dissociation time of 300 s in the assay buffer alone. Global fitting of the interaction curves was performed using the Octet Data analysis software to determine binding affinities (equilibrium dissociation constant, K_D_).

### 2.3. Vaccine Formulations

DTaP-mIPV and DTaP-vIPV contain inactivated poliovirus (IPV) from MRC-5 and Vero cell sources, respectively. The production-scale lots of DTaP-mIPV and DTaP-vIPV were manufactured by Sanofi Limited, Toronto, ON, Canada. Both vaccines are formulated with 6 μg/mL of PRN and contain the same amounts of pertussis toxoid, filamentous hemagglutinin, fimbriae types 2 and 3, diphtheria toxoid, tetanus toxoid, and aluminum phosphate adjuvant. The production-scale lots of lyophilized Act-Hib (*Haemophilus b* conjugate vaccine) were manufactured by Sanofi, Marcy L’Étoile, France. To generate DTaP-IPV/Hib vaccine, DTaP-IPV vaccine was used to reconstitute the lyophilized Act-Hib as per product monograph.

For the heat stress studies, DTaP-vIPV was heated to 60 °C for 1, 2, 4, 7, 14, 21, and 28 days prior to testing.

For use in the assay validation studies, lab-scale mock formulations of DTaP-vIPV were prepared containing the same antigen content, buffer components, and AlPO_4_ adjuvant as the production-scale vaccine, with the exception of PRN. PRN (adjuvanted with AlPO_4_) was then spiked into the mock DTaP-vIPV to generate vaccines with varying levels of PRN antigen.

### 2.4. Common Epitope Screening Test (CEST)

The sera from thirty random individual human donors were obtained from Innovative Research (IPLA-SERS). Equal volumes of sera from the five donors with the highest anti-PRN titers (as determined by an in-house ELISA) were pooled for use in a CEST assay. As the donors were random, the high anti-PRN titers in these donors was presumed to be a result of recent vaccination or recent environmental exposure to *B. pertussis*. In the CEST assay, the ability of this human serum pool to inhibit the binding of mAbs to the purified PRN was assessed. The ELISA plates were coated overnight with PRN (0.6 μg/mL in a carbonate/bicarbonate buffer (Sigma) (Oakville, Ontario, Canada), overnight at 4 °C) and blocked with a wash buffer (PBS, 0.1%Tween 20, 37 °C) containing 1% goat serum (Sigma 16210–064) for 45 min at 37 °C. The pool of high-titer human serum was then added to selected wells (1/5 dilution in a wash buffer/10% goat serum, 1 h at 37 °C). Next, anti-PRN mAb (30 μg/mL in a wash buffer/10% goat serum, 1 h at 37 °C) was added to one well with and one well without the human serum. The binding of mAbs to PRN was detected using HRP-conjugated anti-mouse Ig (1:90,000 dilution, 1 h at 37 °C), and the OD of each well was measured after the addition of 3,3′,5,5′-tetramethylbenzidine (TMB) followed by a H_2_SO_4_ stop solution. The background signal from the wells containing no PRN mAbs and no serum was subtracted from all wells. The reduction in OD signal after the preincubation of PRN with the human serum is reflective of human serum immunoglobulins blocking the binding site for individual anti-PRN mAbs, thus indicating the presence of human antibodies in the serum, which bind the same or overlapping epitopes as the murine mAbs. The experiments were performed five times for each mAb, and the reduction was determined by calculating (OD with serum/OD without serum × 100). As a negative control, an unrelated mAb (anti-HIV mAb clone RP24) was used. 

### 2.5. Serum Inhibition Assay (SIA)

The SIA was a complementary to the CEST and assessed the ability of the mAbs in blocking the binding of human serum antibodies to PRN. The pooled human sera with high anti-PRN titer (see CEST assay) were used in the SIA. PRN (1.0 μg/mL in a carbonate/bicarbonate buffer (Sigma)) was used to coat a 96-well microtiter plate overnight at 4 °C. The plate was blocked for 1 h at 37 °C with a wash buffer (PBS; 0.1%Tween 20) and 1% goat serum (Sigma 16210-064), then individual anti-PRN mAbs (15 µg/mL in an assay buffer [10% goat serum/wash buffer]) were added to selected columns on the plate. As a negative control, an unrelated anti-HIV mAb (clone RP24) was used. 

On each plate, three columns did not receive any anti-PRN mAb, two columns were used as a reference human serum (Human AB Serum, Sigma H4522) (Oakville, Ontario, Canada), and the remaining column was used for determining the titer of the pooled high-titer anti-PRN human sera. The high-titer anti-PRN serum was added to the plate starting at 1/5 dilution, with 3-fold serial dilution down each column for a total of 8 serum dilution points All incubations were 1 h at 37 °C. The plates were washed three times after each step (PBS; 0.1% Tween 20). A goat anti-human HRP conjugate (Invitrogen A24476) (1:90,000 dilution) was added for 30 min, followed by color development with a TMB reagent, and the chromogenic reaction was stopped by the addition of 2N H_2_SO_4_. The plate was read on a spectrophotometer (absorbance at 450nm–absorbance at 540 nm). The anti-PRN titer of the high-titer serum pool was determined by a comparison of its 4-PL response curve to the reference serum curve. The assay was repeated five times independently, and a pair-wise analysis was performed to determine the difference between the serum titer with and without mAb preincubation. The difference in titer was determined for each mAb in each assay, and the mean of the differences was calculated, including the standard error of the mean. The difference in titer between the conditions with and without a specific mAb was considered significant if the standard error of the mean of the differences did not include zero. 

### 2.6. PRN Antigenicity ELISA

The 96-well microtiter plates were coated for 17 to 72 h with anti-PRN mAb 3–5 (2 μg/mL) at 4 °C. The vaccine samples were treated with citrate (1M tri-sodium citrate solution added at 15% of sample volume) at 50 °C for 1.5 h, and the samples were then centrifuged to collect the supernatant fraction. The coated plates were washed three times (PBS; 0.1% Tween 20) and blocked with an assay buffer (PBS; 0.1% Tween 20, 0.1% BSA) for one hour at room temperature. The citrate-treated vaccine supernatant samples and the purified PRN reference standard were each diluted to contain a final concentration of 1 μg/mL of PRN using an assay buffer. The 1 μg/mL PRN reference standard was arbitrarily assigned as 1 Antigenicity Unit per mL (AU/mL). Two independent replicates of each sample and reference standard were prepared per plate, and 3-fold serial dilutions were loaded onto the plate to generate a series of 8 data points per column. After one hour, the plate was washed, and anti-PRN mAb 3–4 HRP conjugate (1:125,000 dilution) was added for 1 h at room temperature. The substrate solution TMB was subsequently added, and the chromogenic reaction was stopped after 15 min by the addition of 2N H_2_SO_4_. Color development was quantified by measuring the absorbance of each well at a wavelength of 450 nm with a reference wavelength of 540 nm using a Spectramax plate reader (Molecular Devices, San Jose, CA, USA)

### 2.7. ELISA Data Analysis

A prerequisite for reporting relative potency is a formal assessment of parallelism between the sample and the reference standard [13]. During the early PRN antigenicity ELISA development, non-parallelism was assessed by using F-Test probability (whereby a F-probability value ≥ 0.05 shows insufficient evidence of non-parallelism), in conjunction with determining that the slope ratio between the sample and the reference standard curves is within 0.8 to 1.25. 

For the test method validation of the antigenicity ELISA, an alternative and preferred approach using equivalence testing to show parallelism between the sample and the reference standard curves [13] was implemented. For this, the 4-PL data generated in the ELISA were analyzed using the SoftMax Pro (6.5.1) software with the Relative Potency module. Parallelism assessment through equivalence testing [13] was performed by determining whether the 90% confidence intervals of a) the slope ratio (B parameter) and b) the ratio of the upper asymptotes (D parameter) of the sample and the reference standard curves are within the equivalence margins, which were provisionally set from 0.67 to 1.50 and from 0.50 and 2.00, respectively. Only upon confirming parallelism was the relative potency of each sample determined (compared to reference standard) and used to calculate a reportable value for PRN antigenicity in arbitrary ‘Antigenicity’ units per mL (AU/mL). 

### 2.8. Method Validation

As per ICH Q2 (R1) guidelines [14], the PRN antigenicity ELISA test method is a quantitative test for a major component. Accordingly, the validation of this assay for DTaP-IPV and DTaP-IPV/Hib vaccines was performed by assessing the parameters of accuracy, precision (repeatability and intermediate precision), specificity, linearity, and range. 

Six mock DTaP-vIPV samples containing 0, 2, 4, 6, 8, and 10 µg/mL of PRN antigen were prepared and designated as samples Q1, Q2, Q3, Q4, Q5, and Q6, respectively, representing a range from 0% to 167% of the nominal PRN concentration used in DTaP-vIPV. The mock vaccine sample Q4 contained the normal amount of PRN used to formulate commercial DTaP-IPV. 

A comprehensive test method validation schedule was designed, using multiple analysts across multiple days (Supplementary Table S1). The six mock DTaP-vIPV samples containing varying PRN concentrations were used to assess accuracy, precision (repeatability and intermediate precision), specificity, linearity, and range. In addition, four representative production-scale lots of DTaP-mIPV, DTaP-vIPV, DTaP-mIPV/Hib, and DTaP-vIPV/Hib were used in the further assessment of assay intermediate precision and repeatability (Supplementary Table S2).

### 2.9. Acellular Pertussis Mouse Immunogenicity Assay

For the acellular pertussis mouse immunogenicity assay, studies were conducted following the established internal routine procedure used by Sanofi. In brief, groups of ten CD-1 mice were each immunized via the intraperitoneal route with a 0.5 mL dilution of a selected DTaP-IPV formulation. The mice were bled 4 weeks post-immunization to measure their serum antibodies against PRN using an ELISA and to identify the number of responding mice per study group.

Animal studies to assess the immune response to heat-stressed DTaP-vIPV were conducted by the Quality Control Bioresources group at Sanofi Limited, Toronto, ON, Canada. 

## 3. Results

### 3.1. Selection of Anti-PRN Monoclonal Antibodies for ELISA Development

Based on previous studies showing their binding to a relatively high proportion of *B. pertussis* cells compared to other anti-PRN mAbs, their ability to inhibit PRN-coated beads from attaching to host cells, and their distinct epitopes [11,12] (Table 1), the anti-PRN mAbs 3–4 and 3–5 were identified as the potential candidates for developing an in vitro potency assay for PRN antigen. Additional characterization of an anti-PRN mAb panel, including these two candidates, was performed to confirm the most appropriate antibodies to proceed with.

Using a biolayer interferometry (BLI), the affinity of the anti-PRN mAbs to the purified PRN was determined. Both mAbs 3–4 and 3–5 had the highest affinities for the PRN antigen among the mAbs tested (Table 1), supporting their use in in vitro assay development over the other anti-PRN antibodies. 

**Figure 1:**
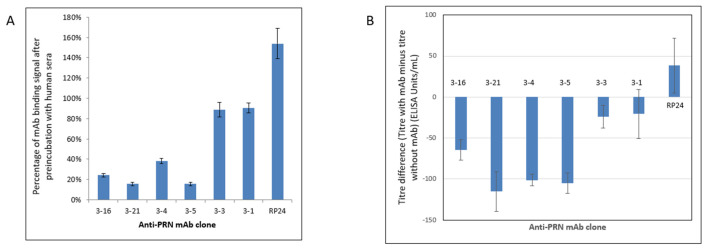
(**A**) Common epitope screening test (CEST) for anti-PRN mAbs. A reduction in optical density (OD) signal after the preincubation of PRN with human serum containing high anti-PRN titers is reflective of antibodies in the sera being capable of blocking the binding site for an individual anti-PRN mAb. Four out of the six anti-PRN mAbs (mAbs 3–4, 3–5, 3–16, and 3–21) have greatly reduced binding to PRN, which had been preincubated with the pooled human sera containing high titers of anti-PRN antibodies. An unrelated non-PRN specific antibody, mAb RP24, serves as a negative control. The reported OD values are shown after the subtraction of OD values from the same samples that did not receive preincubation of human serum. The experiments were performed five times for each mAb. (**B**) Serum inhibition assay (SIA) was used to assess the ability of different anti-PRN monoclonal antibodies in blocking the binding of the high-titer anti-PRN human sera to the PRN antigen. Four anti-PRN mAbs (3–4, 3–5, 3–16, and 3–21) are able to notably inhibit the binding of PRN by the high-titer human sera. An unrelated non-PRN specific antibody, mAb RP24, serves as a negative control. The experiments were performed five times for each mAb.

To further demonstrate the relevance of using mAbs in an in vitro assay, their relation to anti-PRN antibody responses in humans was assessed by examining the ability of the pooled human sera, which contained high titers of anti-PRN antibodies (resulting from vaccination and/or natural exposure to *B. pertussis*), in inhibiting the binding of each mAb to the purified PRN. In this novel common epitope screening test (CEST), a reduction in optical density (OD) signal after the preincubation of PRN with human serum is reflective of human serum immunoglobulins blocking the binding site for an individual anti-PRN mAb. In turn, this indicates the presence of human serum antibodies, which bind the same, or overlapping, epitopes as in the specific murine mAb. The results show that four out of the six anti-PRN mAbs (mAbs 3–4, 3–5, 3–16, and 3–21) have greatly reduced binding to PRN, which had been preincubated with the pooled human sera containing high titers of anti-PRN antibodies (Figure 1A and Table 1).

A serum inhibition assay (SIA) was also performed to confirm the CEST results. In the SIA, the purified PRN was first preincubated with individual candidate anti-PRN mAbs and then incubated with the pooled high-titer anti-PRN human sera. The results showed the same four mAbs (3–4, 3–5, 3–16, and 3–21) were able to inhibit the high-titer anti-PRN human sera from binding PRN (Figure 1B and Table 1). Overall, both the CEST and SIA results provided evidence that these four anti-PRN mAbs bind the same, or overlapping, PRN epitopes as antibodies present in human serum containing high titers of anti-PRN antibodies, thus reinforcing the immunological relevance of these mAbs for consideration in in vitro assay development.

Based on the collective results from our current (Table 1) and previous studies [11,12], mAbs 3–4 and 3–5 were selected to proceed with the PRN antigenicity ELISA development. Both of these mAbs had high affinity, bound unique epitopes (conformational epitope for 3–4, and likely conformational epitope for 3–5), had high relative binding to *B. pertussis* bacteria, showed evidence of relevance to human immunological responses to PRN antigen, and exhibited inhibitory effects on the attachment of PRN-coated beads to the host cells (Table 1) [11,12].

### 3.2. Development of a PRN Antigenicity ELISA 

**Figure 2:**
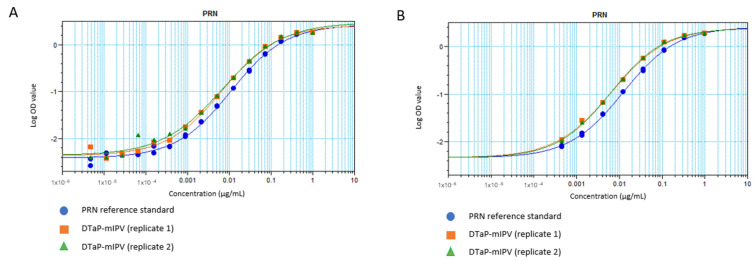
4–PL response curves for the PRN antigenicity ELISA using (**A**) 16–point and (**B**) 8–point dilutions, shown in logarithmic scale. The blue circles show the purified unadjuvanted PRN reference standard. The orange squares and green triangles show the replicate DTaP-mIPV samples.

A sandwich ELISA strategy with mAb 3–5 acting as the capture antibody and mAb 3–4 conjugated to horseradish peroxidase (HRP) as the detection antibody was used. Purified unadjuvanted PRN was selected as the reference standard, as it is amenable to long-term frozen storage, thus contributing to the consistency of the assay. The coating and detecting antibody concentrations were optimized so that the 4-parameter logistic (4-PL) curves, generated by using 16-point serial dilutions of the reference standard (starting at 1 µg/mL and arbitrarily assigned as 1 Antigenicity Unit (AU)/mL), exhibit the upper and lower asymptotes (Figure 2A, blue line). Since the most diluted PRN reference standard points were consistently at background signal levels, the anchoring of the curve to the median OD of the ELISA blank wells was performed for the 8-point reference standard (starting at 1 µg/mL PRN with 3-fold dilutions) for the remainder of the assay development (Figure 2B, blue line). A DTaP-mIPV sample was included, which was treated with citrate to desorb vaccine antigens from the AlPO_4_ adjuvant. The results also show that the vaccine sample (green and orange lines in both Figure 2A,B) presents parallelism to the reference standard that consists of the purified PRN (blue lines). 

**Figure 3:**
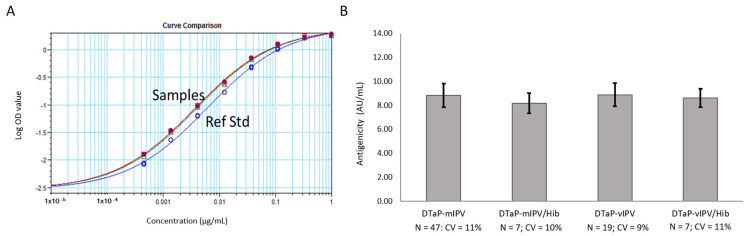
PRN antigenicity ELISA performed on the DTaP-mIPV, DTaP-vIPV, DTaP-mIPV/Hib, and DTaP-vIPV/Hib vaccine matrices. (**A**) 4-PL response curves for the PRN reference standard (blue line, open circles) and each vaccine matrix (remaining overlapping lines). (**B**) PRN antigenicity results for each of the four different vaccine matrices obtained from multiple runs, by multiple analysts, over multiple days. The error bars represent one standard deviation.

Additional studies using the DTaP-IPV vaccines containing IPV from MRC-5 (mIPV) and Vero (vIPV) cell sources, as well as the DTaP-IPV mixed with lyophilized Act-Hib (to form DTaP-IPV/Hib), were conducted. The 4-PL data generated in the ELISA was analyzed using the SoftMax Pro software (version 6.5.1.) Relative Potency module. With each of the four vaccine samples exhibiting parallelism to the reference standard (Figure 3A for representative example), the relative potency of each sample compared to the reference standard was used to calculate a reportable value of arbitrary AU/mL. The results show that the PRN antigenicity values for the DTaP-IPV and DTaP-IPV/Hib samples are very similar (averaging around 8 to 9 AU/mL), thus demonstrating that the reconstituted Act-Hib and the source of IPV (MRC-5 or Vero cells) used to formulate the vaccines did not interfere with the assay (Figure 3A,B). In addition, the results obtained from three different analysts testing the same sample lots over multiple days demonstrate that the PRN antigenicity ELISA has low variability (Figure 3B).

**Figure 4:**
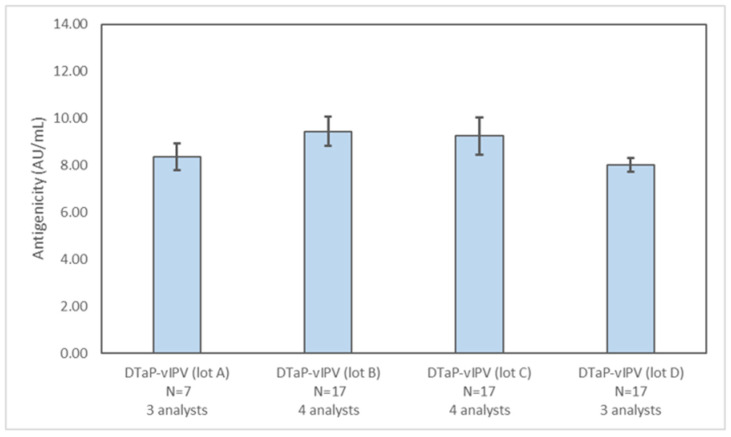
Assessing lot-to-lot variability of PRN antigenicity results in different DTaP-vIPV lots. The PRN antigenicity results for four different DTaP-vIPV lots were obtained from multiple runs, by at least three analysts, over multiple days. The error bars represent one standard deviation.

Multiple different lots of DTaP-vIPV (four lots, Figure 4) and DTaP-mIPV (nine lots, Supplementary Figure S1) were tested with the PRN antigenicity ELISA to assess the utility of the assay for monitoring lot-to-lot consistency. The PRN antigenicity results for multiple lots of both vaccines are similar, confirming the overall consistency of PRN antigenicity in different DTaP-IPV vaccine batches. 

Although the vaccine sample and the reference standard were both diluted in the ELISA to start at the same PRN concentration (based on µg/mL), the vaccine sample response curves are consistently shifted to the left of the reference standard curve (see Figure 3A for an example). This results in the reported antigenicity value (in AU/mL) for the DTaP-IPV and DTaP-IPV/Hib samples being consistently greater than the protein concentration value of PRN used for vaccine formulation (6 μg/mL). To confirm that the ELISA was specific for PRN antigen, a mock vaccine was formulated containing all the antigens, adjuvant, and buffer components found in DTaP-vIPV, with the exception of the PRN antigen. As expected, the ELISA did not detect any PRN signal in the mock vaccine (Supplementary Figure S2), demonstrating that the test method was specific for PRN in DTaP-IPV, and that other antigens, buffer components, or adjuvant of the vaccine did not contribute to PRN signal. In addition, it was demonstrated that the citrate treatment procedure used for antigen desorption (including 50 °C heat treatment and addition of citrate, for 1.5 h) had no effect on the antigenicity measured in the purified PRN antigen alone (Supplementary Material, Figure S3).

### 3.3. Assessing Stability-Indicating Ability of the PRN Antigenicity ELISA 

**Figure 5:**
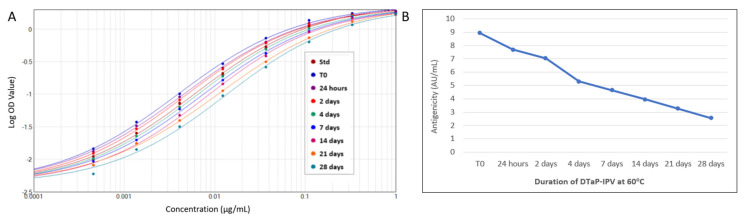
Stability indication of PRN antigen using the PRN antigenicity ELISA. DTaP-mIPV vaccine was heat treated at 60 °C for 0, 1, 2, 4, 7, 14, 21, and 28 days to degrade the PRN antigen. The rightward shift of the response curves with increasing heat-treatment duration (**A**) indicates a decrease in PRN antigenicity over time (**B**).

The ability of the PRN antigenicity ELISA to be used as a stability-indicating assay was assessed by testing the DTaP-mIPV vaccine that was heat treated at 60 °C for 0, 1, 2, 4, 7, 14, 21, and 28 days to degrade the PRN antigen. As shown in Figure 5A, there is a clear rightward shift in the ELISA response curves for the samples that were exposed to increasing duration of heat treatment, resulting in a steady decline in PRN antigenicity over the course of the study (Figure 5B) and, thus, demonstrating the assay is stability indicating.

### 3.4. Method Validation

Validation of the PRN antigenicity ELISA was performed by assessing the parameters of accuracy, precision (repeatability and intermediate precision), specificity, linearity, and range. 

The ICH Q2 (R1) guidance defines the accuracy of an analytical procedure as the closeness of agreement between the value which is accepted either as a conventional true value or an accepted reference value and the value found [14]. As the PRN antigenicity ELISA provides a quantitative measure of intact specific epitopes on PRN in the vaccine sample, there was no established accepted value for PRN antigenicity to assess accuracy. As such, the expected PRN antigenicity value in DTaP-IPV (normally formulated with 6 μg/mL of total PRN protein) was set at 8.81 AU/mL, based on test development data generated across multiple days by multiple analysts on five different representative production lots of DTaP-vIPV (N = 61 data points).

Six mock DTaP-vIPV samples containing 0, 2, 4, 6, 8, and 10 µg/mL of PRN antigen (designated as samples Q1, Q2, Q3, Q4, Q5, and Q6, respectively) were prepared to assess the method validation parameters following a comprehensive testing schedule (Supplementary Table S1). The mock vaccine sample Q4 contained the normal amount of PRN used to formulate DTaP-IPV and, thus, represented the 100% level. Four representative production lots of DTaP-mIPV, DTaP-vIPV, DTaP-mIPV/Hib, and DTaP-vIPV/Hib were also tested to provide additional data for determining the tested ELISA’s intermediate precision and repeatability.

For accuracy assessment, the ratio of the geometric mean of the measured antigenicity to the expected antigenicity for PRN was calculated for each concentration level of the mock DTaP-vIPV samples. The average recovery for PRN antigenicity at each PRN concentration level is between 0.8 to 1.2 (inclusive), meeting the acceptance criteria for method accuracy (Table 2).

Repeatability and intermediate precision of the method were assessed using three concentration levels of the mock DTaP-vIPV samples (Q2, Q4, and Q6) and with one concentration level of each of the production samples (DTaP-mIPV, DTaP-vIPV, DTaP-mIPV/Hib, and DTaP-vIPV/Hib). All coefficients of variation (%CV) for repeatability and intermediate precision are less than 15%, meeting the specified acceptance criteria (%CV ≤ 15%) (Table 3). 

**Figure 6:**
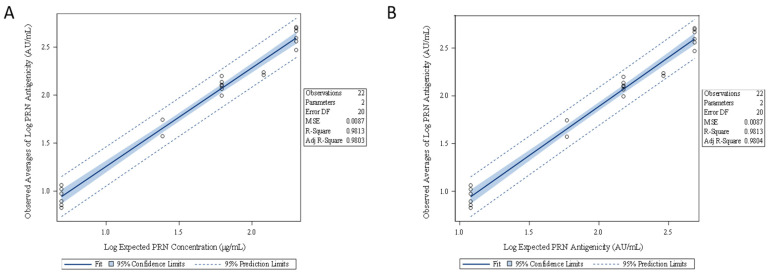
Linearity assessment of the PRN antigenicity ELISA from five concentration levels of PRN in DTaP-vIPV. Linear regression analysis was performed using (**A**) the observed averages of log PRN antigenicity (AU/mL) vs. log expected PRN concentration (µg/mL), and (**B**) the observed averages of log PRN antigenicity (AU/mL) vs. log expected PRN antigenicity (AU/mL).

The linearity of the ELISA was assessed by using the data generated from five concentration levels (Q2 to Q6) of the mock DTaP-vIPV sample. Method linearity was confirmed by performing a linear regression analysis for (1) the observed averages of log PRN antigenicity (AU/mL) vs. log expected PRN concentration (µg/mL) (Figure 6A), and (2) the observed averages of log PRN antigenicity (AU/mL) vs. log expected PRN antigenicity (AU/mL) (Figure 6B). In either case, the results are linear with R^2^ value greater than 0.98. The specificity of the ELISA was confirmed as there were no valid measurable PRN antigenicity results from the mock DTaP-vIPV sample that contained no PRN antigen. 

The range of the method was determined using the highest and lowest concentrations of PRN antigen that met the acceptance criteria for accuracy, precision, and linearity. As such, the ELISA was validated to test the vaccine samples containing PRN at 2 µg/mL to 10 µg/mL, corresponding to the PRN antigenicity values of 2.56 AU/mL to 13.67 AU/mL, respectively, based on the geometric mean antigenicity values measured for levels Q2 and Q6 used in the assay validation (Table 2).

### 3.5. Comparing the PRN Antigenicity ELISA with the In Vivo Acellular Pertussis Mouse Immunogenicity Assay

Heat-stressed DTaP-IPV formulations (60 °C over a period of 28 days) were tested with the PRN antigenicity ELISA and with the traditional acellular pertussis mouse immunogenicity assay to assess the impact of heat stress on PRN protein. The same degraded DTaP-IPV used in the ELISA test (Figure 5) was injected into mice in two separate studies.

There was no clear indication of decreasing anti-PRN sera titers in either animal study (Table 4). In contrast, the same heat-treated DTaP-IPV tested in the PRN antigenicity ELISA demonstrated a progressive drop in antigenicity over time (Figure 5B and Table 4). These results show that the PRN antigenicity ELISA is more sensitive in detecting alterations to PRN antigen compared to the animal assay. 

## 4. Discussion

In this study, an ELISA was developed and validated to provide a measure of antigenicity for PRN in DTaP-IPV and DTaP-IPV/Hib vaccines. Antigenicity describes the ability of a molecule (e.g., protein antigen) to be recognized at specific epitopes by the antibodies generated from an immune response to that molecule. The development of in vitro assays to replace or to reduce the use of in vivo assays for routine vaccine testing requires a comprehensive characterization of relevant reagents, such as monoclonal antibodies. As such, identifying relevant antibodies that recognize the key epitopes involved in antigen functionality, stability, and/or other important characteristics that produce effective immune responses are the key for such an in vitro assay development.

Based on comprehensive studies conducted on a panel of anti-PRN mAbs, two candidate antibodies were selected with the most relevant qualities for use in the antigenicity ELISA. These qualities included their ability to bind to a relatively high proportion of *B. pertussis* cells, their ability to inhibit PRN-coated beads from attaching to host cells, their high affinity and recognition of distinct PRN epitopes, their ability to detect degraded PRN, and evidence of their relevance to human responses.

Although the vaccine sample and reference standard were both diluted to the same starting PRN concentration in this ELISA, their response curves do not necessarily overlap. This is not unexpected, as the antigenicity ELISA is based on comparing the PRN epitope availability in highly complex vaccine matrices to a much simpler non-adjuvanted purified PRN reference standard. Importantly, the ELISA showed consistent parallelism between the reference standard and the vaccine samples, had good precision and linearity, demonstrated specificity for PRN antigen, and was stability indicating. The established precision and linearity of this ELISA also gave further support of the accuracy of this test method, which was in alignment with the ICH Q2 (R1) guidelines as well [14].

As highlighted in Ph. Eur. 5.2.14, a demonstration of agreement between in vitro and in vivo assays is not necessarily expected [15]. One-to-one comparison between in vivo and in vitro methods can be challenging since antigenicity assays are usually more sensitive than in vivo assays, and they examine a specific element of potency, such as the presence of intact, functionally important epitopes on the vaccine antigen. It is, however, expected that the antigenicity assay preferably reflects both antigen content and integrity of the antigen by targeting relevant epitopes [15]. The in vitro test should be capable of detecting antigen alteration/differences, and, therefore, samples that differ in the magnitude of response should be used. By testing heat-stressed DTaP-IPV samples, it was evident that the PRN antigenicity ELISA was superior to the animal-based potency test in detecting changes in the PRN antigen. The mouse immunogenicity assay is less sensitive in part due to the recognized variability of animal tests in general (Table 4 results, as example). Another limitation of this animal test is that any mouse antibodies generated against degraded PRN may still bind to the coating antigen in the ELISA used to determine anti-PRN sera titers, thus giving titer values that still show a strong response. Due to the sensitivity of the PRN antigenicity ELISA compared to the in vivo mouse immunogenicity assay, it offers an advantage with respect to the monitoring of consistency of the manufacturing process and may be more relevant for assessing the impact of vaccine manufacturing changes. Furthermore, animal tests are known to be highly variable, and, as concluded in a recent survey of in vivo potency tests for DTaP vaccines, this variability limits their value for their intended purpose [16]. 

When introducing alternative in vitro tests to replace animal tests, the lack of defined acceptance criteria can be a challenge [1]. Future application of the PRN antigenicity ELISA would align with the ‘consistency approach’. This approach is based upon a thorough characterization of a vaccine using relevant physicochemical and/or immunological analytical methods that generate a ‘product profile’ (including parameters, such as antigenicity, purity, and identity), with the quality of subsequent batches relying on strict application of a quality system to ensure consistent batch production [17]. With sufficient product profile data, the consistency approach can allow for the establishment of vaccine product acceptance criteria without the need for accompanying animal studies. Examples can be found with Human Papillomavirus Virus-like particle (HPV-VLP) vaccines and pneumococcal polysaccharide conjugate vaccines, where release testing relies on validated animal-free methods assessing batch-to-batch consistency [17]. The PRN antigenicity ELISA for DTaP-IPV provides an opportunity to implement the consistency approach into more established ‘legacy’ vaccine production. 

Efforts to better characterize candidate mAbs and/or to assess in vitro assays for vaccine testing (including DTaP vaccines) have been undertaken by different groups [18,19,20], including the Vaccine to Vaccine (VAC2VAC) project managed by the Innovative Medicines Initiative 2 (IMI2) [21]. The key objective of the VAC2VAC was to provide proof of concept of the consistency approach for batch release testing of established vaccines through the development of in vitro analytical methods [21]. The characterization of anti-PRN mAbs summarized in this, and other studies [11,12], and the development of a PRN antigenicity assay that involves comparison to an actual animal potency test helps to further the strategies used for developing relevant in vitro assays.

Based on these studies, the PRN antigenicity ELISA is proposed as a possible in vitro replacement of the in vivo potency test for PRN in DTaP-IPV-based vaccines. Important considerations, including comprehensive antibody characterization, testing of multiple lots, method validation, and demonstration of higher discriminative power than the in vivo test, were used to support the relevance of this in vitro assay, which can be applied in future strategies to replace animal testing.

## Figures and Tables

**Table 1:** Summary of the characterization data for the anti-PRN monoclonal antibodies.

Anti-PRN mAb Clone	Isotype	Affinity (K_D_; nM)	Epitope Identification by HDX (Amino Acid Position on PRN) ^1^	Epitope Type	mAbs Binding to *B. pertussis* Surface ^2^	Relevance to Human Response (CEST)	Relevance to Human Response (SIA)	Inhibition of PRN-Coated Bead Binding to Host Cells ^3^
3–1	IgG1	0.143	Not tested	Not tested	<25%	NO	NO	0.7
3–3	IgG1	12.2	588–591	Linear/Continuous	<25%	NO	NO	0.1
3–4	IgG1	0.23	420–436, 455–464, 477–492, and 505–517	Conformational	>25%	YES	YES	0.7
3–5	IgG1	0.51	234–244	Likely conformational (formed by linear sequence)	>25%	YES	YES	0.4
3–16	IgG1	6.68	36–46 and 56–67	Conformational	>25%	YES	YES	0.1
3–21	IgG1	16.5	35–78 and109–116	Conformational	>25%	YES	YES	0.4

^1^ Data from reference [12]. ^2^ Data from reference [11] showing the percentage of positively stained bacteria as determined by flow cytometry. ^3^ Data from reference [11] showing relative bead-binding prevention capacity.

**Table 2:** Accuracy of the PRN antigenicity ELISA from the test method validation.

Sample	PRN Concentration (µg/mL)	Expected PRN Antigenicity (AU/mL)	Number of Data Points (N)	Geometric Mean of Measured Antigenicity (AU/mL)	Average Recovery	Acceptance Criteria (0.8 ≤ Recovery ≤ 1.2)
Q2	2	2.94	18	2.56	0.9	Satisfied
Q3	4	5.87	6	5.25	0.9	Satisfied
Q4	6	8.81	18	8.16	0.9	Satisfied
Q5	8	11.74	6	9.23	0.8	Satisfied
Q6	10	14.68	18	13.67	0.9	Satisfied

**Table 3:** Repeatability and intermediate precision of the PRN antigenicity ELISA from the test method validation.

Sample	N	Repeatability		Intermediate Precision	
		%CV Repeatability	Acceptance Criteria (% CV ≤ 15%)	Intermediate Precision (% CV)	Acceptance Criteria (% CV ≤ 15%)
Q2 (2 µg/mL PRN)	18	5%	Satisfied	10%	Satisfied
Q4 (6 µg/mL PRN)	18	6%	Satisfied	9%	Satisfied
Q6 (10 µg/mL PRN)	18	7%	Satisfied	11%	Satisfied
DTaP-mIPV/Hib	6 ^1^, 12 ^2^	9%	Satisfied	11%	Satisfied
DTaP-mIPV	6 ^1^, 12 ^2^	12%	Satisfied	11%	Satisfied
DTaP-vIPV/Hib	6 ^1^, 12 ^2^	6%	Satisfied	9%	Satisfied
DTaP-vIPV	6 ^1^, 12 ^2^	4%	Satisfied	9%	Satisfied

^1^ For assessing repeatability. ^2^ For assessing intermediate precision.

**Table 4:** PRN mouse immunogenicity and antigenicity ELISA results on heat-treated DTaP-IPV samples.

	PRN Mouse Immunogenicity Study 1		PRN Mouse Immunogenicity Study 2		PRN Antigenicity ELISA Results
Treatment	GMU Fold ^1^	# of Responders Meeting Criteria ^2^	GMU Fold ^1^	# of Responders Meeting Criteria ^2^	Antigenicity (AU/mL)
Time zero	2.5	Yes	5.1	Yes	8.95
24 h, 60 °C	5.5	Yes	3.4	Yes	7.72
2 days, 60 °C	5.4	Yes	3.5	Yes	7.05
4 days, 60 °C	2.6	Yes	4.3	Yes	5.32
7 days, 60 °C	6.9	Yes	4.6	Yes	4.64
14 days, 60 °C	4.5	Yes	8.2	Yes	3.96
21 days, 60 °C	4.8	Yes	3.2	Yes	3.26
28 days, 60 °C	5.1	Yes	2.6	Yes	2.56

^1^ Geometric mean unitage (antibody titer) is shown as a fold increase over the minimum passing GMU acceptance criteria. Any value equal to, or greater than 1, indicates the GMU has met the acceptance criteria. ^2^ Indicates whether the number of responding mice met/exceeded the minimum acceptance criteria.

## Data Availability

Data sharing is not applicable to this article.

## Supplementary Materials

The following supporting information can be downloaded at https://www.mdpi.com/article/10.3390/vaccines11020275/s1, Figure S1: Assessing lot-to-lot variability of PRN antigenicity results in different DTaP-mIPV lots tested once by single analyst; Figure S2: Confirming specificity of the PRN antigenicity ELISA by testing a mock DTaP-vIPV sample lacking PRN antigen (blue line) compared to purified PRN reference standard (red line) and mock vaccine spiked with nominal amount of PRN (green line); Figure S3: Antigenicity of Untreated and Citrate/Heat treated Purified PRN Antigen; Table S1: PRN Antigenicity ELISA validation study testing schedule using mock DTaP-IPV spiked with varying concentrations of PRN; Table S2: PRN Antigenicity ELISA validation study testing schedule using different production scale vaccines.
