# Supplementary material for: Development of an In Vitro Test Method to Replace an Animal-Based Potency Test for Pertactin Antigen in Multivalent Vaccines
Source: Vaccines (Basel). 2023 Jan 27;11(2):275. doi: 10.3390/vaccines11020275 (PMC9965796; doi:10.3390/vaccines11020275)
Supplement: Supplementary file 1 [file vaccines-11-00275-s001.zip › vaccines-2133347-supplementary.pdf]

Supplementary Figures

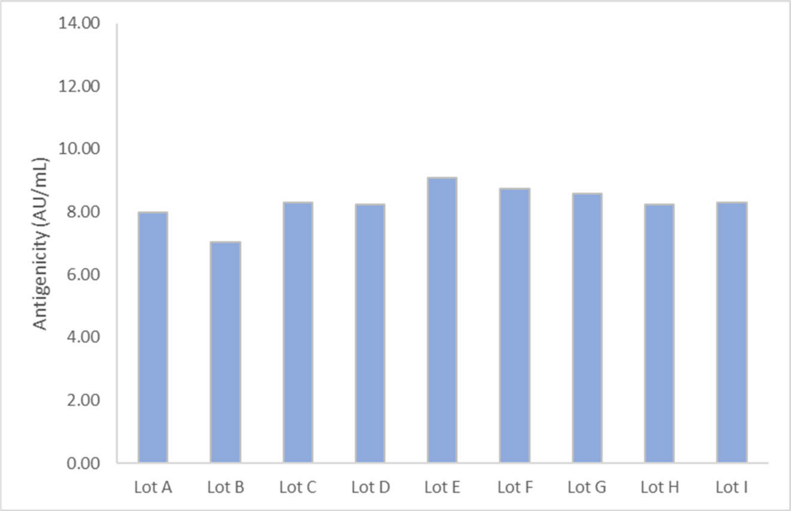

Supplementary Figure S1. Assessing lot-to-lot variability of PRN antigenicity results in different DTaP-mIPV lots tested once by single analyst. The nine different DTaP-mIPV lots are arranged in order of manufacturing date, and were formulated over the span of approximately 18 months.

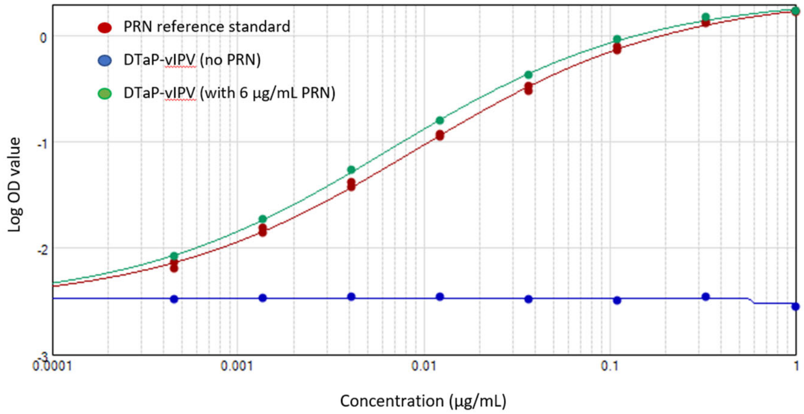

Supplementary Figure S2. Confirming specificity of the PRN antigenicity ELISA by testing a mock DTaP-vIPV sample lacking PRN antigen (blue line) compared to purified PRN reference standard (red line) and mock vaccine spiked with nominal amount of PRN (green line).

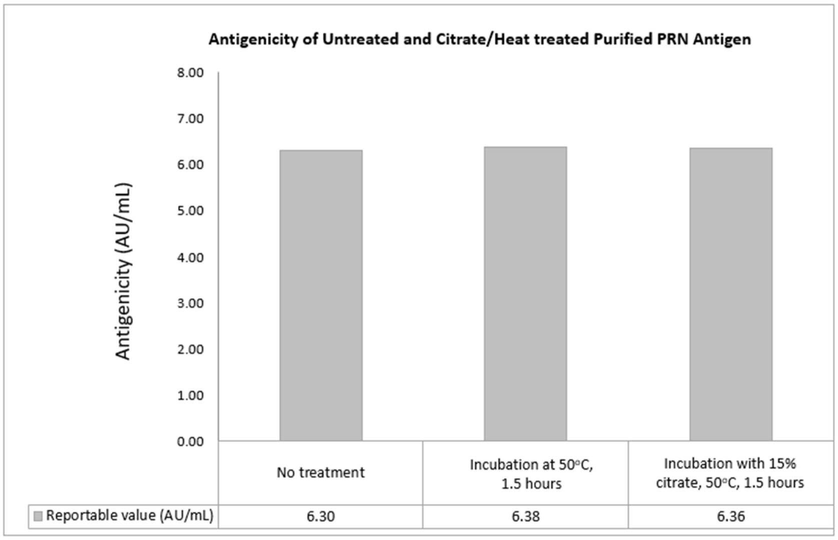

Supplementary Figure S3. Assessing antigenicity of purified PRN antigen treated with, or without, the citrate (1M citrate added at 15% sample volume) and heat (50°C) used in the desorption process.

Supplementary Table S1. PRN Antigenicity ELISA validation study testing schedule using mock DTaP-IPV spiked with varying concentrations of PRN.

Sample	Total Data Points Obtained	Number of Analysts	Day of testing	Number of data points obtained per day, per analyst [1]	Validation parameter assessed
**Q1**	6	2	2 days	3	Specificity
**Q2**	18	2	6 days	3	Accuracy, Intermediate Precision, Repeatability Linearity, Range
**Q3**	6	2	2 days	3	Accuracy, Linearity, Range
**Q4**	18	2	6 days	3	Accuracy, Intermediate Precision, Repeatability, Specificity, Linearity, Range
**Q5**	6	2	2 days	3	Accuracy, Linearity, Range
**Q6**	18	2	6 days	3	Accuracy, Intermediate Precision, Repeatability Linearity, Range

[1] Each of the three data points generated for a specific sample on a specific day were obtained from independent runs.

Supplementary Table S2. PRN Antigenicity ELISA validation study testing schedule using different production scale vaccines.

Sample	Total Data Points Obtained	Number of Analysts	Validation parameter assessed
**DTaP-mIPV**	6[1], 12[2]	4	Repeatability, Intermediate Precision
**DTaP-mIPV/Hib**	6[1], 12[2]	4	Repeatability, Intermediate Precision
**DTaP-vIPV**	6[1], 12[2]	4	Repeatability, Intermediate Precision
**DTaP-vIPV/Hib**	6[1], 12[2]	4	Repeatability, Intermediate Precision

[1] For assessing repeatability, six independent runs were performed on one day by one analyst. [2] For assessing intermediate precision, twelve independent runs were performed over 3 days, by four analysts each day.
